# Improved performance of small molecule solar cell by using oblique deposition technique and zinc phthalocyanine cathode buffer layer†

**DOI:** 10.1039/c8ra01136b

**Published:** 2018-03-19

**Authors:** Tianjiao Zhao, Gengmin Zhang, Yingjie Xing

**Affiliations:** Key Laboratory for the Physics and Chemistry of Nanodevices, Department of Electronics, Peking University Beijing 100871 China xingyj@pku.edu.cn

## Abstract

Short circuit current density (*J*_sc_) and open circuit voltage (*V*_oc_) are two important parameters to evaluate the performance of organic solar cells. How to increase these two parameters without using novel material still remains a challenge. Two small molecules, zinc phthalocyanine (ZnPc) and 3,4,9,10-perylene tetracarboxylic bisbenzimidazole (PTCBI), are used to fabricate ITO/ZnPc/ZnPc:PTCBI/PTCBI/ZnPc/Al photovoltaic device. We find that *J*_sc_ and *V*_oc_ are enhanced by using oblique deposition technique and ZnPc cathode buffer layer, respectively. Analysis of the active layer reveals phase segregation in obliquely deposited ZnPc:PTCBI bulk heterojunction layer. Field emission measurement is used to probe the band bending and internal field in ZnPc/PTCBI planar heterojunction. The effects of phase segregation and internal field are discussed. This work shows that careful assembly of donor and acceptor material is beneficial to small molecule photovoltaic device.

## Abstract 2

*J*_sc_ and *V*_oc_ of ZnPc:PTCBI bulk heterojunction solar cell are enhanced by using oblique deposition technique and ZnPc cathode buffer layer.

## Graphical abstract

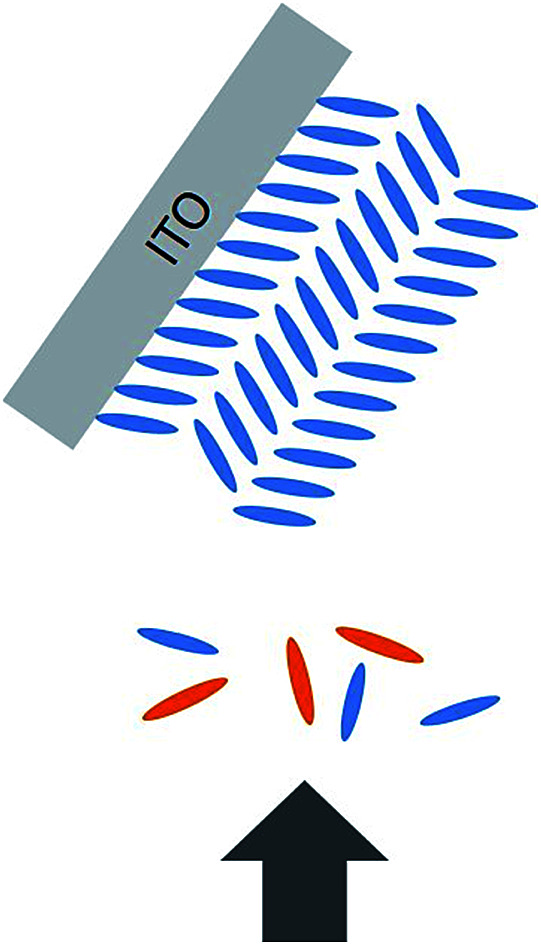

## 1 Introduction

Organic photovoltaic cells have shown great potential as a means to transform solar energy to electricity. Research efforts in the last two decades have significantly improved organic solar cell performance, such as high short circuit current density (*J*_sc_), large open circuit voltage (*V*_oc_), and good fill factor (FF). As a result, a record efficiency larger than 11% is achieved in bulk heterojunction (BHJ) organic solar cells.^1^ However, some intrinsic problems hamper further improvement of BHJ organic solar cells, *e.g.*, charge losses in donor:acceptor BHJ and a trade-off between the external quantum efficiency (EQE) and the voltage loss between the optical gap and *V*_oc_.^2,3^ A reduced driving force for charge separation is measured in many donor–acceptor systems with small energy level offset, although a different conclusion is given with non-fullerene acceptor recently.^4–6^

BHJ structure in small molecule solar cell can be prepared by co-evaporation of donor and acceptor molecules, and the donor:acceptor BHJ layer is usually sandwiched between a pure donor layer and a pure acceptor layer, which is called p–i–n structure. It has been found that by introducing proper phase separation in the BHJ layer, the charge losses are reduced significantly, and bimolecular recombination is not considered as a severe problem in BHJ photovoltaic devices.^7^ The most efficient approach to create proper phase segregation in metal phthalocyanine:fullerene BHJ is to deposit a template layer on the substrate before co-deposition. Small aggregate nucleates in the blend film due to the interaction between metal phthalocyanine and template molecules. For example, hexaazatriphenylene-hexacarbonitrile (HAT-CN) and copper iodide (CuI) are employed as the template molecule for copper phthalocyanine (CuPc) and zinc phthalocyanine (ZnPc), respectively.^8,9^ Thermal annealing is another common method to produce phase segregation in the BHJ layer.^10^ It seems that the external force outside the donor–accepter system, such as template molecule or annealing treatment, is necessary to create phase separation in small molecule BHJ film.

Besides the active layer, the interfacial buffer also influences the performance of small molecule solar cell. For example, a thin layer of organic insulator inserted between the acceptor layer and the cathode blocks the hole transport and eliminates the exciton annihilation at the cathode effectively.^11^ Some semiconducting small molecules and the combination of two materials have also been used as the cathode layer in literature.^12,13^ Up to date, bathocuproine (BCP) is still the most often used cathode buffer insulator in small molecule solar cell. Other roles of the cathode buffer layer include protection from the damage during the cathode metal deposition, optical spacer for modulating the optical field, and avoiding the air diffusion to the active layer.^11–14^

ZnPc and 3,4,9,10-perylene tetracarboxylic bisbenzimidazole (PTCBI) are among the earliest used small molecules. However, there is no study on ZnPc:PTCBI BHJ device in literature. We use ZnPc and PTCBI to fabricate ITO/ZnPc/ZnPc:PTCBI/PTCBI/buffer/Al device. The present work is the first report on ZnPc:PTCBI BHJ solar cell to the best of our knowledge. We show that *J*_sc_ and *V*_oc_ of ZnPc:PTCBI photovoltaic device can be increased without other material or external treatment. Our result shows that the efficiency of organic solar cell can be improved by proper assembly of donor and acceptor material in both crystalline grain scale and the active layer scale based on their intrinsic properties.

## 2 Material and methods

### 2.1 Device fabrication and measurement

**Fig. 1:**
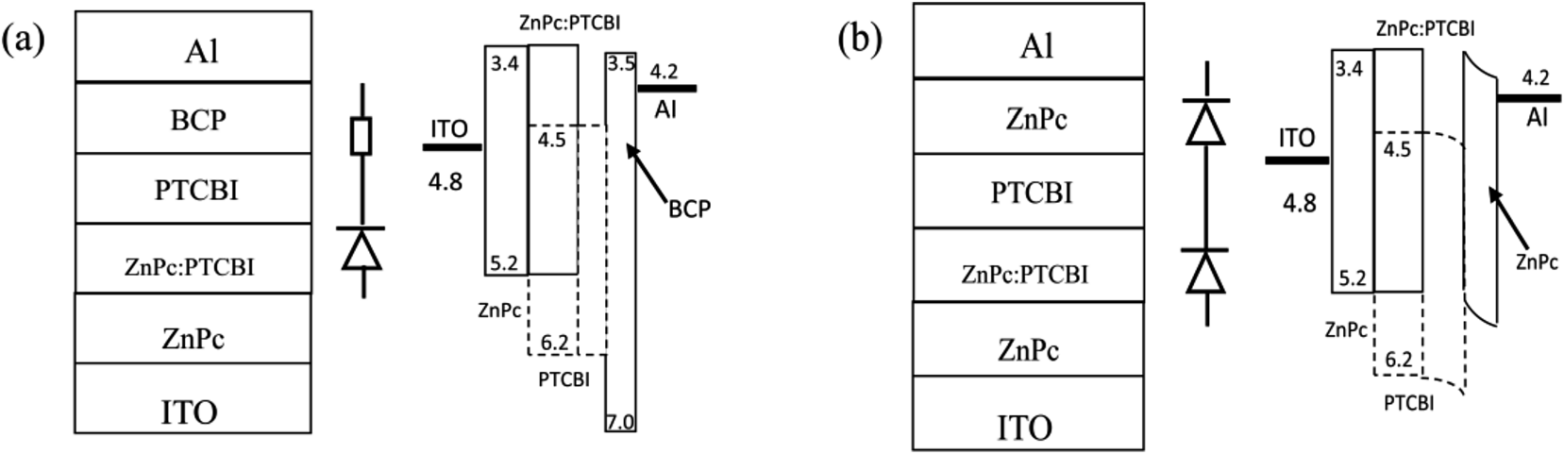
Device structure, schematically device model, and proposed band diagram of photovoltaic device with BCP buffer (a) and ZnPc buffer (b).

Cleaned indium tin oxide (ITO) glass was used as the substrate in all devices. ZnPc (Yannuo Chem., purity 99%), PTCBI (Yannuo Chem., purity 99%), and bathocuporine (Yannuo Chem., purity 99%) were used as received. The configuration of all devices was ITO/ZnPc/ZnPc:PTCBI/PTCBI/buffer/Al. Either BCP or ZnPc was used as the buffer material in photovoltaic device. Fig. 1 shows the device structure and the proposed energy level diagram. The ZnPc : PTCBI mixtures were all in weight ratio 1 : 1.5, which was optimized by many experiments. The devices were fabricated in a commercial vacuum deposition system (ULVAC-KIKO VWR-400M/ERH). Organic materials were deposited onto ITO substrates successively at a rate of ∼0.05 nm s^−1^ under a pressure of ∼8 × 10^−4^ Pa. Aluminium was deposited at a rate of ∼0.5 nm s^−1^ under a pressure of 5 × 10^−3^ Pa. The thicknesses of ZnPc (15 nm)/ZnPc:PTCBI (24 nm)/PTCBI (17 nm)/buffer (8 nm)/Al (100 nm) layers were used in all devices. The film thickness and deposition rate were monitored *in situ* using a quartz crystal oscillator. The area of a single device was 4 mm^2^. At least three batches of photovoltaic devices were prepared under each condition. The best efficiency in each batch was compared in this work. All samples were annealed at 110 °C for half an hour in vacuum. A sunlight simulator (Newport Oriel 91160) was used to illuminate the sample with the power of 100 mW cm^−2^. The current–voltage curves were measured by an electrochemical analyzer in air. Typical *J*–*V* curves were simulated to extract the device parameters according to a one-diode model.^15^ Optical absorption was measured with a UV-Vis-NIR spectrophotometer (Cary 5000) in air. Monochromatic incident photon-to-electron conversion efficiency (IPCE) analysis was carried out in air (Newport 74125). Atomic force microscope (AFM, DI NanoScope) was used to scan the surface of organic film. Transmission electron microscope (TEM, FEI Tecnai F30) was used to observe nanoscale morphology of organic films.

### 2.2 Oblique angle deposition

**Fig. 2:**
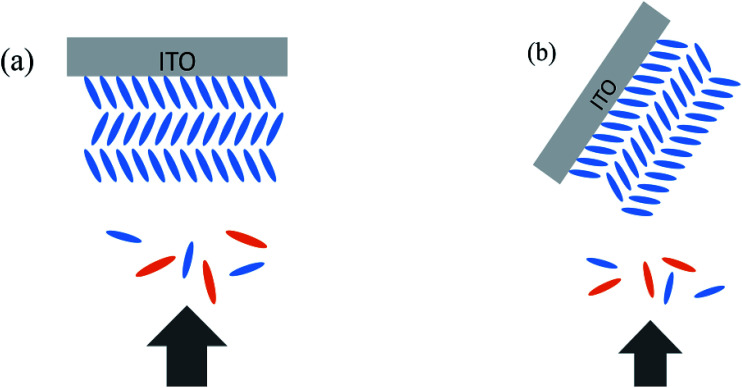
Schematically demonstration of deposition of ZnPc:PTCBI BHJ on edge-on ZnPc grains. (a) Perpendicular direction, (b) oblique deposition. ZnPc: blue, PTCBI: orange.

Both perpendicular deposition and oblique angle deposition were used in our experiment. Perpendicular deposition means the evaporant flux reaches the substrate perpendicularly (shown in Fig. 2a). In oblique angle deposition (shown in Fig. 2b), we tilted the substrate before the deposition, and ZnPc/ZnPc:PTCBI/PTCBI layers were deposited on the substrate obliquely. Then the substrate was tuned back to the normal horizon position in the vacuum chamber *in situ* by manual operation. Perpendicular deposition was used to deposit buffer/Al layers to complete the device. We note the oblique angle in our experiment is different from nearly 90° in previous reports of glancing angle deposition, by which metal phthalocyanine columnar structure was formed *via* a self-shadowing effect.^16^

The oblique degree of the substrate in our experiment is ∼55°, which is shown in Fig. 2b schematically. The reason for choosing this angel is given in ref. 17 and the ESI.† To determine the actual thickness of organic layers deposited obliquely, controlled samples were fabricated and observed by scanning electron microscopy (SEM). Typical thicknesses were calibrated in this way and used in this work. Total thickness of ZnPc/ZnPc:PTCBI/PTCBI layers kept the same value for all devices, which was 56 nm approximately. We think the possible thickness difference due to different deposition angle can be excluded in this way.

Four types of photovoltaic devices were fabricated in this work. The information of all devices is summarized in Table 1. Among all measured samples, the highest efficiency of device A and the intermediate efficiency of device B, C, and D, are listed in Table 1.

**Table 1:** Photovoltaic performance and extracted parameters of devices

Device no.	*J*_sc_ (mA cm^−2^)	*V*_oc_ (V)	FF	PCE (%)	*R*_s_ (Ω cm^2^)	*R*_sh_ (Ω cm^2^)	*n*
A: perpendicular + BCP	0.84	0.41	0.50	0.17	83.4	1942	1.54
ITO/ZnPc/ZnPc:PTCBI/							
PTCBI/BCP/Al							
B: oblique + BCP	1.71	0.41	0.50	0.34	37.7	925	1.58
ITO/ZnPc/ZnPc:PTCBI/							
PTCBI/BCP/Al							
C: perpendicular + ZnPc	0.96	0.53	0.43	0.22	55.6	1173	1.49
ITO/ZnPc/ZnPc:PTCBI/							
PTCBI/ZnPc/Al							
D: oblique + ZnPc	2.40	0.52	0.46	0.57	28.4	709	1.89
ITO/ZnPc/ZnPc:PTCBI/							
PTCBI/ZnPc/Al							

### 2.3 Field emission measurement

A home-made field emission microscope was fabricated to investigate the band bending in organic–organic heterojunction. *In situ* deposition and field emission measurement were conducted in sequence for several times to probe the thickness dependence of work function. The base pressure of field emission microscope was 2 × 10^−7^ Pa. Each layer had a thickness of 1–2 nm. The total thickness of the organic layer on top of the base W tip was ∼15 nm. We found that field emission from the fresh surface was quite stable without the influence of absorption. The voltage applied on the tip was limited to make sure a small and stable emission current (<5 × 10^−6^ A). The detail of the field emission measurement can found in ref. 18 and the ESI.†

## 3 Results and discussion

### 3.1 Effect of oblique angle deposition

**Fig. 3:**
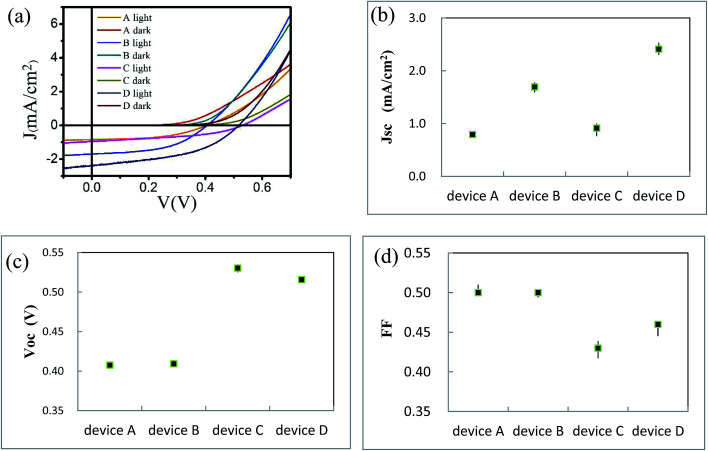
(a) *J*–*V* curves of device A, B, C, and D; statistic result of *J*_sc_ (b), *V*_oc_ (c) and FF (d) of device A, B, C, and D.

We use BCP as the buffer material and fabricate ITO/ZnPc/ZnPc:PTCBI/PTCBI/BCP/Al device at the perpendicular position (denoted as device A). We try different thicknesses of the active layer with this device structure. The highest efficiency is listed in Table 1 and the measured current density–voltage (*J*–*V*) curve is plotted in Fig. 3a. The performance of device A is compared to ZnPc/PTCBI photovoltaic device because no result of ZnPc:PTCBI BHJ device is found in the literature.^19,20^ Similar *V*_oc_ but smaller *J*_sc_ are measured in BHJ device (device A) comparing to planar heterojunction device. This result shows that the pristine BHJ structure is not a good choice for ZnPc-PTCBI system.

We have reported the improvement of *J*_sc_ in ZnPc:C60 BHJ device prepared by oblique deposition.^17^ Here, we use the same technique to fabricate the first three ZnPc/ZnPc:PTCBI/PTCBI layers. BCP and alumina layers are deposited perpendicularly in series to complete the device (denoted as device B). *J*–*V* curve of device B is shown in Fig. 3a and the parameters of device B are listed in Table 1. Comparing to device A, device B shows similar *V*_oc_ and FF but larger *J*_sc_. The statistical data of *J*_sc_, *V*_oc_ and FF of all devices are shown in Fig. 3b–d. A doubled efficiency of device B shows the benefit of oblique deposition on ZnPc:PTCBI BHJ.

**Fig. 4:**
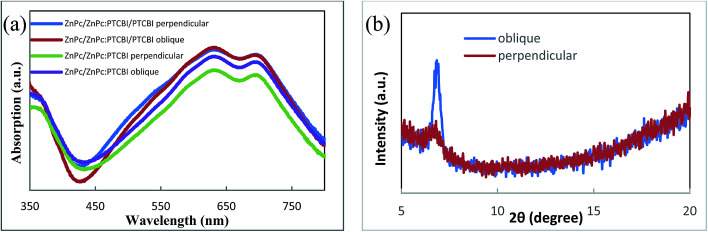
(a) Optical absorption spectra and (b) XRD spectra of samples deposited perpendicularly and obliquely.

Optical measurement and morphology observation are used to investigate the reason for the higher *J*_sc_. We first compare the optical absorption of samples deposited under two conditions. Because BCP/Al bilayer is the same in device A and B, ZnPc/ZnPc:PTCBI/PTCBI triple layer are deposited on the quartz substrate for comparison. The optical absorption spectra are shown in Fig. 4a. Similar optical absorption in a wide wavelength range is observed in two samples. Two peaks at 632 and 692 nm come from the Q-band absorption of ZnPc.^21^ The contribution of PTCBI absorption can be found from a unobvious shoulder at 533 nm in these samples.^22^ Similar optical absorption seems contradictory to the higher *J*_sc_ of device B. Further optical measurement of ZnPc/ZnPc:PTCBI bilayer sample reveals more absorption in the obliquely deposited sample (shown in Fig. 4a). This phenomenon means more photons are absorbed in the obliquely deposited ZnPc:PTCBI BHJ layer under the same illumination.

**Fig. 5:**
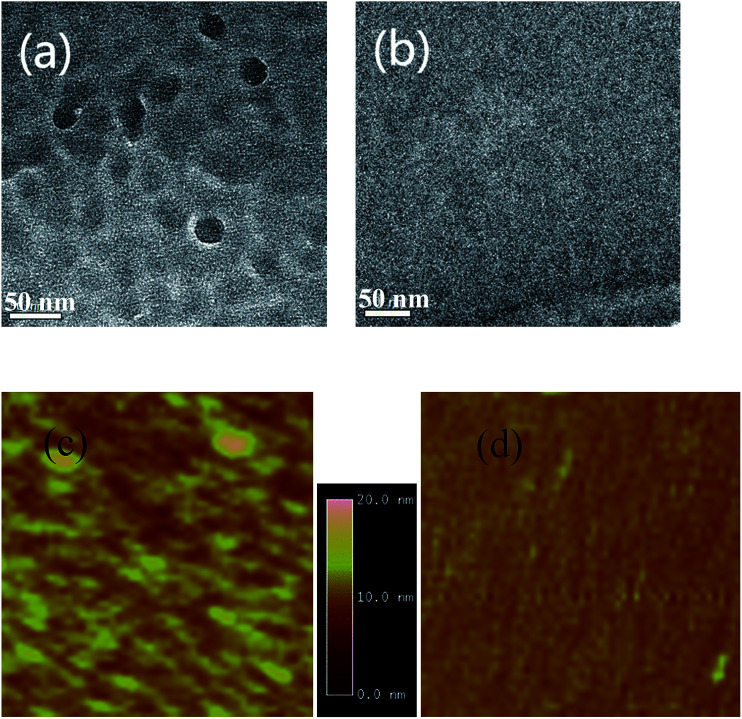
Nanomorphology of ZnPc:PTCBI BHJ. (a) TEM and (c) AFM image of obliquely deposited sample, (b) TEM and (d) AFM image of perpendicularly deposited sample. The area of square (c) and (d) is 1 μm × 1 μm.

XRD is also used to investigate the crystalline structure of BHJ samples. ZnPc/ZnPc:PTCBI bilayer are deposited on ITO substrate under two conditions and the measured result is shown in Fig. 4b. No obvious peak is found in the XRD spectrum of perpendicularly deposited sample. However, the XRD spectrum of obliquely deposited sample shows a sharp peak at 6.8°, which is the indicator of monoclinic (200) plane of α-ZnPc grains.^23^ This result indicates that oblique deposition produces a large number of crystalline ZnPc grains in ZnPc:PTCBI BHJ. TEM and AFM are employed to observe the nanomorphology of ZnPc:PTCBI BHJ directly. ZnPc/ZnPc:PTCBI bilayer are deposited on a copper grid covered by a porous carbon film for TEM observation. Some small particles are found in the BHJ layer prepared by oblique deposition (shown in Fig. 5a), whereas in the BHJ film deposited at the perpendicular position, a featureless surface is observed (shown in Fig. 5b). The particles in Fig. 5a have the size of approximately twenty nanometers and distribute in the BHJ film randomly. This morphology difference clearly reveals phase segregation in ZnPc:PTCBI BHJ deposited obliquely. AFM investigation also shows different nanomorphology of two BHJ samples. RMS roughnesses of 3.40 and 2.65 nm are measured in BHJ deposited obliquely and perpendicularly (shown in Fig. 5c and d), respectively. Larger RMS roughness by AFM scanning also confirms the appearance of phase separation in obliquely deposited BHJ film.^24^

Based on above analyses, we conclude that phase segregation occurs in obliquely deposited ZnPc:PTCBI BHJ layer, and higher *J*_sc_ is the result of phase segregated BHJ layer. A brief explanation for phase segregation is given here and in the ESI.† The growth of ZnPc film and the orientation of ZnPc grains have been studied detailedly.^25^ ZnPc grains have two stacking orientations (shown in the ESI, Fig. S1†): edge-on (standing) and face-on (lying). It has been found that phase segregation occurs in the BHJ layer deposited on face-on ZnPc grains but not on edge-on ZnPc grains, because the formation and growth of ZnPc nuclei is less disturbed by the acceptor molecules on face-on ZnPc grains.^9^ However, edge-on ZnPc grains always grow on bare ITO substrate. Therefore, in order to induce phase segregation in BHJ, CuI is used as the template molecule to grow the face-on ZnPc grains firstly.^9^ In our experiment, device A is fabricated at perpendicular position (shown in Fig. 2a). An intimate mixture BHJ on edge-on ZnPc grains is the product after co-evaporation of ZnPc and PTCBI. When we tilt the substrate of device B, two facets of edge-on ZnPc grain are exposed to co-evaporated ZnPc and PTCBI flux (shown in Fig. 2b). The side facet of edge-on ZnPc grain acts like the top surface of face-on ZnPc grain, resulting in the phase separation in ZnPc:PTCBI BHJ.

We extract diode parameters from *J*–*V* curve to probe the influence of phase segregation on charge transport.^15^ The result reveals that charge transport in obliquely deposited ZnPc:PTCBI BHJ is improved. The diode parameters are listed in Table 1. Device B shows a much lower *R*_s_ (37.7 Ω cm^2^) than device A (83.4 Ω cm^2^). A smaller shunt resistance (*R*_sh_) in device B may be the result of higher charge density in BHJ layer under irradiation.^26^

### 3.2 Effect of ZnPc cathode buffer layer

One problem limiting *V*_oc_ in organic solar cell is a large voltage loss between the optical gap and *V*_oc_ (>0.6 V).^4,5^ Reduction of this voltage loss can be realized by optimizing the energy level offset between donor and acceptor. However, this method usually brings a new problem of weakening the driving force for charge separation. We try to enlarge the open circuit voltage in a different way. The position of *V*_oc_ is defined at the zero-current point, where the photocurrent equals to the injection current. This definition means that if the photocurrent does not change, *V*_oc_ will be enlarged when we depress the injection current intentionally.^27^

Organic semiconductor/metal Schottky junction have been studied for a long time.^28–30^ Here, we use ZnPc as the buffer material instead of BCP to fabricate ITO/ZnPc/ZnPc:PTCBI/PTCBI/ZnPc/Al device (denoted as device C). Perpendicular deposition is used to prepare device C. A Schottky diode inside device C is formed due to ZnPc/Al contact. We expect a lower injection current in device C, because the current through two series-connected diodes is lower than that through one diode under the same positive bias. The dark current of device C is lower than that of device A under the same bias (shown in Fig. 3a), confirming the effect of ZnPc/Al junction. The parameters of device C are listed in Table 1 and the statistical data of *V*_oc_ is shown in Fig. 3c. An enlargement of *V*_oc_ (0.12 V) due to ZnPc buffer layer is measured as expected. The schematically device model is shown in Fig. 1, in which BCP acts as a resistor in device A, whereas ZnPc/Al contact behaves like an additional diode in device C. *J*_sc_ of device C is a little larger than that of device A. We suggest that the larger *J*_sc_ comes from the optical spacer effect of ZnPc buffer, which has been observed in other buffer materials.^31,32^ Although both *V*_oc_ and *J*_sc_ are enlarged by ZnPc buffer in device C, the improvement of efficiency is not obvious (∼30%). This performance is influenced by the lower FF of device C, because the *J*–*V* curve is deviated intentionally by the depressed injection current.

Different from BCP buffer in device A, conducting ZnPc buffer in device C produces an additional PTCBI/ZnPc planar heterojunction in device C. Then a confusing problem emerges: is there an opposite-direction photocurrent in PTCBI/ZnPc heterojunction under illumination? If some excitons dissociate at the planar PTCBI/ZnPc interface, an opposite-direction photocurrent will appear in PTCBI/ZnPc heterojunction according to the concentration gradient mechanism.^33^ A consequent result of this opposite-direction photocurrent is to cancel part of the photocurrent generated in ZnPc:PTCBI BHJ layer. However, we do not observe a decreased *J*_sc_ in device C, reveling no or negligible effect of the opposite-direction photocurrent. We propose two reasons to explain this phenomenon: weak optical absorption and internal field. Because of the similar band gap of ZnPc (1.9 eV) and PTCBI (1.7 eV), the optical absorption in the pure PTCBI layer is less than that in ZnPc/ZnPc:PTCBI bilayer and the least absorption appears in the ZnPc layer underneath Al cathode. Therefore, the number of excitons generated in PTCBI/ZnPc heterojunction is much less than those generated in ZnPc:PTCBI BHJ.

**Fig. 6:**
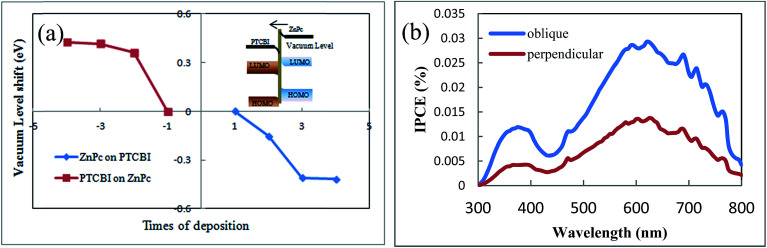
(a) Vacuum level shift in ZnPc/PTCBI heterojunction measured by field emission, inset: band bending and internal field in ZnPc/PTCBI heterojunction. (b) IPCE spectra of device C and D.

The second reason for no or negligible opposite-direction photocurrent is the internal field in PTCBI/ZnPc heterojunction, which comes from the electron transport across PTCBI/ZnPc interface under equilibrium condition. We propose that this internal field is against the opposite-direction photocurrent. Although some theories are proposed to describe the charge transfer across the organic–organic interface, such as the integer charge transfer (ICT) model and the induced density of interface states (IDIS) mechanism,^34,35^ there still lacks a well-developed model to predict the actual electronic structure in organic–organic heterojunction up to date. Directly measuring the band structure by photoelectron spectroscopy is the most often used method in literature.^36,37^ We develop a method to detect the band bending in organic–organic heterojunction.^18^ This method employs the sensitivity of field emission current to the work function of sample tip. The band bending in PTCBI/ZnPc heterojunction is calculated from the variation of the slope of linear Fowler–Nordheim plot. The detail of the field emission measurement can be found in ref. 18 and the ESI.† The band bending diagram reveals an internal field in the direction from ZnPc to PTCBI (shown in Fig. 6a). This direction is in accordance with the result of ZnPc/PTCBI measured by electroabsorption.^38^ Such an internal field hinders the concentration gradient driven diffusion of electrons and holes away from the PTCBI/ZnPc interface. We note there are other mechanisms to influence the charge transport in organic heterojunction, and this internal field is just one possible reason to explain the negligible opposite-direction photocurrent in device C.

Based on the results of oblique deposition and ZnPc buffer layer, we fabricate ITO/ZnPc/ZnPc:PTCBI/PTCBI/ZnPc/Al device (denoted as device D), in which ZnPc/ZnPc:PTCBI/PTCBI triple layer are deposited obliquely and ZnPc/Al bilayer are deposited perpendicularly. The measured *J*–*V* curve is shown in Fig. 3a. Similar to device C, larger *V*_oc_ appears in device D due to the effect of ZnPc/Al junction. Device D shows the highest efficiency and *J*_sc_ as expected. The benefit of phase separation in ZnPc:PTCBI BHJ is proved by lower series resistance in device D (28.4 Ω cm^2^) than that in device C (55.6 Ω cm^2^). Monochromatic incident photon-to-electron conversion efficiency (IPCE) measurement of device D confirms a better performance in all wavelength (shown in Fig. 6b).

FF of device D (0.46) is a little higher than that of device C (0.43). This result is verified by repeated experiments and the statistical data is shown in Fig. 3d. Because the only difference between device C and D is the substrate angle, we suggest that phase separation is the reason for the higher FF of device D. Considering the effect of the internal field due to PTCBI/ZnPc contact, the extraction of photo-generated carriers from ZnPc:PTCBI BHJ should be enhanced. Because charge recombination and loss behavior is different in phase separated BHJ and in intimate mixing BHJ,^7^ the extraction of charge carrier is sensitive to the degree of phase separation in ZnPc:PTCBI BHJ. FF value stands for a factor how efficient the photo-generated carriers are extracted from the photovoltaic device. The higher FF of device D means more efficient charge transport and less charge loss in phase separated ZnPc:PTCBI BHJ. This phenomenon is in accordance with some recent studies on the relation between FF value and the degree of phase separation in BHJ solar cells.^39,40^ We think this additional effect of the cathode buffer layer can be used to help the charge extraction in other phase separated BHJ device.

## 4 Conclusions

In conclusion, small molecule BHJ solar cells based on ZnPc and PTCBI are fabricated. *J*_sc_ and *V*_oc_ are improved by using oblique deposition technique and ZnPc cathode buffer layer, respectively. Phase separation occurs in obliquely deposited ZnPc:PTCBI BHJ. The internal field in PTCBI/ZnPc planar heterojunction shows two effects: reducing the opposite-direction photocurrent in PTCBI/ZnPc heterojunction and helping the charge extraction in ZnPc:PTCBI BHJ. Our result shows that the performance of small molecule solar cell can be improved by considering the stacking style of small molecules and the internal filed in donor/acceptor heterojunction.

## Conflicts of interest

There are no conflicts to declare.

## Supplementary Material

RA-008-C8RA01136B-s001
