# Supplementary material for: Improved performance of small molecule solar cell by using oblique deposition technique and zinc phthalocyanine cathode buffer layer
Source: RSC Adv. 2018 Mar 19;8(20):10999–1005. doi: 10.1039/c8ra01136b (PMC9078964; doi:10.1039/c8ra01136b)
Supplement: RA-008-C8RA01136B-s001 [file RA-008-C8RA01136B-s001.pdf]

Electronic Supplementary Material (ESI) for RSC Advances.
This journal is © The Royal Society of Chemistry 2018

Supplementary information

Improved performance of small molecule solar cells by using oblique deposition technique and ZnPc cathode buffer layer

Tianjiao Zhao, Gengmin Zhang and Yingjie Xing*

Key Laboratory for the Physics and Chemistry of Nanodevices and Department of Electronics, Peking University, Beijing 100871, China

* xingyj@pku.edu.cn

1 Oblique angle deposition

We already know that face-on ZnPc grains show some capability of promoting the formation of ZnPc nuclei during the co-evaporation of ZnPc and C60, but edge-on ZnPc grains do not have such capability. [1] Therefore, amorphous ZnPc:C60 BHJ forms on edge-on ZnPc grains, whereas phase separation in ZnPc:C60 BHJ occurs on face-on ZnPc grains. Because edge-on ZnPc grains always grow on ITO substrate (shown in Fig. S1a), particular treatment must be proceeded to ITO substrate to grow face-on ZnPc grains (shown in Fig. S1c).

Different from the substrate treatment, we find an alternative way to partly reproduce the face-on "surface" with edge-on ZnPc grains. The angle between edge-on ZnPc molecule and substrate is 64°. When we tune the substrate, some side facets of ZnPc grains will be forced to expose to the evaporant flux. The "surface" of ZnPc grains opposite to the evaporant flux partly looks like the surface of face-on ZnPc grains (shown in Fig. S1b). In this way, the requirement for phase segregation in BHJ is introduced in our experiment without substrate treatment.

The angle of the substrate relative to the incident beam is tuned in the vacuum chamber *in situ* by manual operation. The oblique angle in present experiment is ~

55°, which is close to 64°. We find that in this angle, the substrate location before and after rotation is suitable for both oblique and perpendicular deposition in our evaporation system.

Figure S1a and S1b show two types of deposition schematically. A thin layer of ZnPc is deposited on ITO substrate firstly. Edge-on ZnPc grains grow on both horizontal-placed and tilted ITO substrate (shown in Fig. S1a and S1b). Then ZnPc and PTCBI molecules are evaporated simultaneously. The oblique substrate exposes some similar deposition facets (shown in Fig. S1b) as those in face-on ZnPc (shown in Fig. S1c) except a small deflection (9°), resulting in phase segregation in ZnPc:PTCBI BHJ.

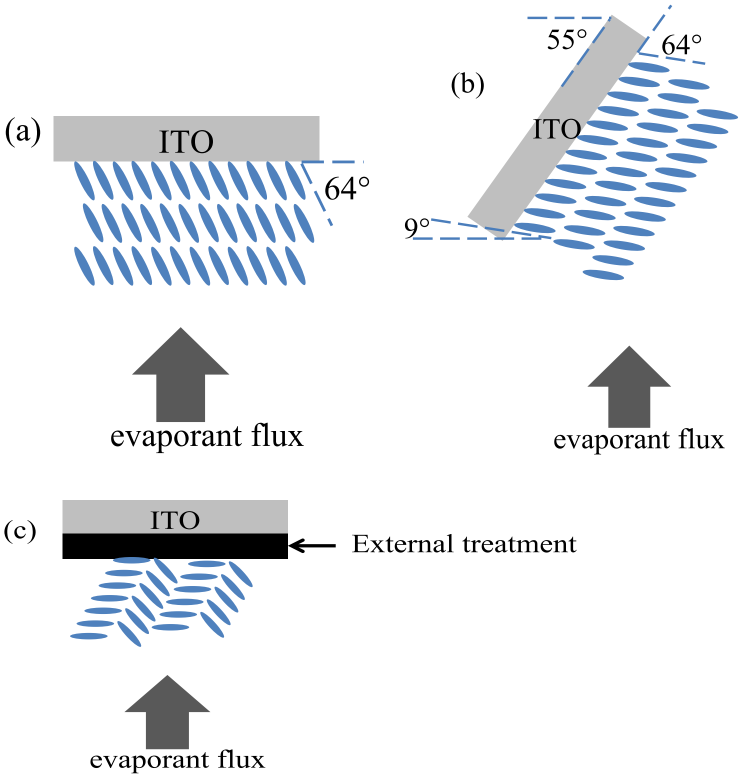

Fig. S1 Schematic demonstration of co-deposition of ZnPc and PTCBI on ZnPc coated ITO substrate, (a) horizontal substrate and (b) tilted substrate. (c) Face-on ZnPc grains grown on particularly treated ITO substrate. Blue ellipse stands for ZnPc molecules.

2 Field emission measurement

2.1 Calculation method

The detail of the field emission measurement can found in Ref. 34. According to Fowler-Nordheim theory, a better approximation for the slope of F-N plot (S), that considers the effect of the imaging force, gives

$$S = -6.83 \times 10^7 \frac{\varphi^{3/2}}{\beta} s(y)$$

where φ is the work function, β is the local field conversion factor, $s(y)$ is a slowly varying function with a value of 1 to 0.833; in first-order approximation, 0.917 may be used. If we assume that the value of β remains constant, variation of the work function ($\Delta\varphi$) is the only reason for a changed slope (ΔS). Then the change of work function can be calculated by comparing the slope of F-N plot using the following Equation (φ and S are initial work function and slope, respectively).

$$\varphi + \Delta\varphi = \left(\frac{S + \Delta S}{S}\right)^{\frac{2}{3}} \varphi$$

In organic-organic heterojunction deposited on a sharp W tip, the change of work function of the sample reflects a small shift of the vacuum level at the sample surface after one time of deposition.

2.2 Result

Fermi levels of ZnPc (3.8 eV) and PTCBI (4.6 eV) are used to calculate $\Delta\varphi$. These values are adopted from Ref. 2 and 3.

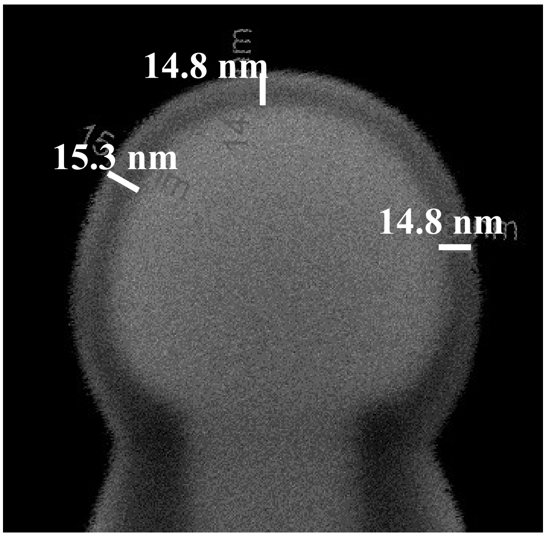

Fig. S2 SEM image of a typical tip after field emission measurement. The semitransparent out layer is the ZnPc/PTCBI bilayer with the thickness of ~ 15 nm, the brighter central part is W-tip.

Table S1. Band bending in ZnPc/PTCBI heterojunction. Δφ is the energy difference between Fermi level and the vacuum level.

Times of deposition (PTCBI on ZnPc)	Slope	$\Delta\varphi$ (eV)	R^2
4	7549	—	0.9966
3	7571	0.009	0.9987
2	7708	0.064	0.9981
1	8621	0.426	0.9920
Times of deposition (ZnPc on PTCBI)	Slope	$\Delta\varphi$ (eV)	R^2
4	6830	—	0.9944
3	6808	0.008	0.9953

2	6108	0.266	0.9936
1	5710	0.416	0.9941

References:

[1] Y. Zhou, T. Taima, T. Miyadera, T. Yamanari, M. Kitamura, K. Nakatsu and Y. Yoshida. Phase separation of coevaporated ZnPc:C60 blend film for highly efficient organic photovoltaics. Appl. Phys. Lett. 100, 233302 (2012).

[2]S. H. Park, J. G. Jeong, H.-J. Kim, S.-H. Park, M.-H. Cho, S. W. Cho, Y.Yi, M. Y. Heo, and H. Sohn, Appl. Phys. Lett. 96, 013302 (2010).

[3] I.G. Hill, J. Schwartz, A. Kahn, Organic Electronics 1 (2000) 5-13
